# Indents

**Published:** 1910-06-15

**Authors:** 


### INDENTS.

☞ **Brief Articles of Technical or Scientific Value will receive notice and comment. Contributions invited.**

*Arsenous Acid in the Painless Devitalization of the Pulp.*

A review of the universal dental literature on devitalization of the pulp by arsenous acid since its introduction by Spooner in 1836 is offered, and a method for the painless devitalization as evolved by eighteen years of clinical experiments is suggested, as follows: A pellet of cotton saturated with concentrated carbolic acid is laid on the exposed pulp, if it has been possible to expose the same, for at least ten minutes. The effect of the arsenous acid is painless only if an arsenous paste thoroughly mixed with carbolic acid is applied. In exposed pulps this paste is brought in to direct contact with the pulp. The paste is applied on cotton, and sealed in the cavity very carefully and without exerting pressure. The greater the volume of the pulp the more paste is required for devitalization. The arsenous paste should be left in the cavity for a certain definite time, which is determined by the kind of tooth and the size of the pulp. If the paste is left in a tooth with a large pulp for over two and one-half days danger of periodontitis arises. The greater the surface upon which the paste can exert its action, the more quickly the devitalization of the pulp will take place. The action of the arsenous acid is a gradual one by way of necrosis of the tissue. The differential diagnosis between simple acute pulpitis, acute purulent pulpitis, acute gangrenous pulpitis and chronic granulomatous pulpitis is of great importance for the correct application of the paste. Dr. Lipschitz's formula for arsenous paste is:

℞—Acid. arsenic.,
Cocain. hydrochlor., āā 1.0
Acid. carbol., q.s. to make a soft paste.

Dr. Lipschitz in *Cosmos*.

*Keeping Impression Trays Clean.* In order to remove wax or plaster from impression trays, and to keep them clean and bright, apply steel wool No. 2 for removing the bulk, and No. 0 for polishing. They will then always look new.—C. V. Campbell in *Cosmos.*

*Preparing Stearate of Zinc.* I get the stearate of zinc in an ounce bottle; it is a very light powder and I spatulate one-third to half of that into an ounce of vaseline on a large plaque after melting the vaseline. I could incorporate a great deal more, but I could not handle it in the syringe. I use the chlorid of zinc with it in severe cases. I have used iodin with it, but I have almost ceased using anything but the stearate of zinc. —J. B. Pherrin in *Review.*

*Ulcerated Tooth Caused Man's Death.* Neglect of an ulcerated tooth, that led to septic poisoning, was given as the cause of the death of Joseph Kratchman, of 227 Holsman street, Paterson, N. J., by members of the family, on February 12th.

Kratchman, who was a silk weaver, first had the tooth attended to two years ago. He then had it filled. A month ago it began to trouble him again, and he went to a dentist to have it cared for. The filling was removed and preparations were made to refill the molar.

This time the man's face began to swell, and after it had been cared for Kratchman went to a physician to have the swelling reduced. By this time the man's condition became so serious that a consulting physician was called in. The swelling had spread to every tooth in the patient's head, and by the orders of the physician Kratchman was taken to the General Hospital.

The poison had spread with such rapidity that the only thing that could be done was to make incisions and drain the wound off and stay further spread of the disease. But the trouble had continued so long that nothing could save the man's life and he died at the hospital.—*Scrap Book.*

*New York Bill in the Interests of Dentistry.* Assemblyman Joseph of New York City, has introduced in the New York Legislature on February 7th a bill that will be productive of great good to the dental fraternity. It deserves commendation and a vote of help from every dental society in the State of New York.

Below is the text of the bill:

An act to amend the penal law, in relation to corporations practising medicine, dentistry or pharmacy.

The People of the State of New York, represented in Senate and Assembly, do enact as follows:

Section 1. Chapter eighty-eight of the laws of nineteen hundred and nine, entitled the "penal law," constituting chapter forty of the consolidated laws, is hereby amended by adding a new section, to be known as section two hundred and eighty-one, to be inserted after section two hundred and eighty, as follows:

Section 281. It shall be unlawful for any corporation to practice medicine, dentistry or pharmacy or make it a business to practice medicine, dentistry or pharmacy or to hold itself out to the public as being entitled to practice medicine, dentistry or pharmacy, or to render or furnish services or advice or to furnish doctors, dentists or pharh macists to render services of any kind, of any nature or in-any other way or manner, or in any other manner to assume to be entitled to practice medicine, dentistry or pharmacy or to assume, use or advertise the title of doctor, dentist or pharmacist or equivalent terms in any language in such a manner as to convey the impression that it is entitled to practice medicine, dentistry or pharmacy, or to furnish advice or services as a doctor, dentist or pharmacist or to advertise that either alone or together, with or by or through any person, whether a duly and regularly licensed doctor, dentist or pharmacist or not, owns, conducts or maintains an office, place of business or store for the practice of medicine, dentistry or pharmacy or for furnishing advice or services as doctor, dentist or pharmacist. Any corporation violating the provisions of this section shall be liable to a fine of not more than five thousand dollars, and every officer, trustee, director, agent or employee of such corporation who directly or indirectly engages in any of the acts herein prohibited, or assists such corporation to do such prohibitive acts, is guilty of a misdemeanor. The fact that any officer, trustee, director, agent or employee shall be a duly and regularly licensed doctor, dentist or druggist shall not be held to permit or allow any such corporations to do the acts prohibited herein, nor shall such fact be a defense upon the trial of any of the persons mentioned herein for a violation of the provisions of this section. This section shall not apply to any corporation lawfully engaged in the business authorized by the provisions of any existing statute, nor shall it prohibit a corporation from employing a duly licensed doctor, dentist or pharmacist in and about its own immediate affairs, nor shall it apply to organizations organized for benevolent or charitable purposes.—*Scrap Book.*

*Aged and Indigent Dentists.* New Jersey is the first and only State in the Union that has inaugurated a systematic method for the relief and care of its aged and indigent members. It is a cause for reproach that men who have unselfishly worked for their chosen profession should want in the waning years of their lives and often become the inmates of alms houses. There is no use preaching a homily on the fact, they should have saved for old age in their youth. If they had been selfish like many others, dentistry would have lost many valuable contributions to its history and its inventions. Barnum, of rubber dam fame, would have been wealthy if he had patented the use of it in dentistry, but he gave it to the profession. Webb would have been better off if he had never cliniced or gone to society meetings, or done anything for the profession's advancement. Any number of cases could be enumerated within the history of dentistry. Now let some of the progressive members of the several States societies, at their meetings during the year, offer resolutions similar to the New Jersey idea, viz.: increase the annual dues $1.00 per year and set the amounts aside as a nucleus to a fund that will grow by bequests and contributions.—*Dental Scrap Book.*

*Dental vs. Medical Requirements.* A dentist to be successful must be a surgeon, an artist, a sculptor, and a mechanic. He must have the same mental grasp of the laws of physics, chemistry, and biology as is needed by the physician. He must have the manipulative skill that is required by the surgeon in his most delicate work. He must be able to take advantage of the finest requirements of the mechanic, and must have the ability to carry out those mechanical operations on living tissue in such manner as to cause no irritation thereto. His workshop is a hole in the face about two inches in diameter; in that hole he has to perform all of his operations, and the patient takes the work away with him.

In nine-tenths of the work done by the physician or surgeon, nature is expected to complete what he leaves. The dentist has to *do* his work. His failures stand out where he can always see them. The doctor buries his. Many deaths of infants are due to the physician's ignorance of the terrible effects of interrupted dentition. Most diseases are greatly aggravated by unsanitary oral conditions that the physician ignores completely, but that every dentist appreciates. I venture the assertion that half the diseases that take toll of mankind will be controlled when dentistry has succeeded in teaching people to keep their mouths clean, and their teeth in condition to masticate their food properly and vigorously. The beauty, vigor and health of the human body and mind are greatly dependent on the possession of a sound, useful masticating apparatus. Is not the man who is able to control this situation worthy of equal honor with the writer of prescriptions? Is he not a bigger man? Does he not deserve more credit? It takes him just as long to acquire his education. His dental course consists of three years of thirty-four weeks each, exclusive of holidays. He has to work from nine to five for six days every week. This course requires more hours' work than is covered by a three-year medical course. The dental student is grounded in the same fundamental subjects, such as anatomy, physiology, chemistry, histology, materia medica, pathology, bacteriology, etc., besides the many special subjects belonging solely to dentistry.

Dentistry gave anesthesia to the world (Wells and Morton both being dentists), and a Cleveland dentist is today teaching the medical profession how prolonged anesthesia can be secured with almost perfect safety without the use of dangerous chloroform or the sickening ether. The medical men, however, attempt to take the honor of anesthesia to themselves, just as they have claimed the honor of bacteriology which was developed by Pasteur, an obscure chemist, whom the medical men were too supercilious to listen to for years.

There is another thing to which I want to direct your attention in connection with the dentist's shop. The man under his care is usually in a bad humor. He does not go to the dentist until he has to, as a rule, and as soon as he gets there he begins to fuss about countless other things he would rather be doing; as a result he gets peevish and will not sit still. The dentist has to show consideration. He must be tolerant; he has to do all the smiling, both for his patient and for himself; his best efforts are seldom appreciated. He is commonly regarded as a disagreeable necessity. His task is a thankless one; and because as a rule he is square and honest, and charges by the hour or by the operation, he does not make as much money as he ought to make. A surgeon can put up a bluff. He can make a mountain out of a mole-hill and charge the price for removing a tumor when he takes out a wart, and the patient will never be any the wiser. The most the physician has to do is to look wise and let nature take her course. Nature has precious little to do with the restoration of teeth in the human mouth.—Elbert Hubbard, *The Era*

